# Respiratory Muscle Rehabilitation in Patients with Prolonged Mechanical Ventilation: A Targeted Approach

**DOI:** 10.1186/s13054-020-2783-0

**Published:** 2020-03-24

**Authors:** Bernie Bissett, Rik Gosselink, Frank M. P. van Haren

**Affiliations:** 1 Discipline of Physiotherapy, University of Canberra, Bruce, ACT Australia; 2 Intensive Care Unit, Canberra Hospital, Garran, ACT Australia; 3 Department of Rehabilitation Sciences, KU Leuven, Health Science Campus Gasthuisberg O&N IV, Leuven, Belgium; 4 Australian National University Medical School, Canberra, ACT Australia; 5 Faculty of Health, University of Canberra, Bruce, ACT Australia

## Abstract

This article is one of ten reviews selected from the Annual Update in Intensive Care and Emergency Medicine 2020. Other selected articles can be found online at https://www.biomedcentral.com/collections/annualupdate2020. Further information about the Annual Update in Intensive Care and Emergency Medicine is available from http://www.springer.com/series/8901.

## Introduction

Early and proactive rehabilitation of intensive care unit (ICU) patients is essential to reverse or minimize the impact of ICU-acquired weakness [1]. While ICU clinicians have largely focused on whole-body exercise to address limb muscle weakness (e.g., early mobilization), we now know that respiratory muscle weakness is twice as prevalent as limb muscle weakness in ICU patients [2]. Moreover, respiratory muscle weakness is associated with a higher risk of extubation failure [3], a longer duration of ventilator-dependence [4] and worse outcomes in terms of hospital mortality [2] and mortality within 1 year [3]. While ventilator-weaning failure is complex, and respiratory muscle weakness is only one contributing factor [5], this weakness is modifiable and can respond to targeted training. In this context, it is surprising that respiratory muscle rehabilitation is not yet standard practice in many ICUs around the world.

Drawing on recent and emerging evidence, we will give an overview of the impact of respiratory muscle weakness in ICU patients (both at the physiological and patient level), and summarize the current evidence regarding the effects of respiratory muscle training. We will also outline strategies for identifying respiratory muscle weakness in ICU patients, as well as an evidence-based and pragmatic approach to providing targeted and individualized respiratory muscle rehabilitation in the ICU. Finally, we will describe the newest technological developments that have radically changed the scope of respiratory muscle rehabilitation for even our most profoundly weak ICU patients.

### Respiratory Muscle Weakness in ICU Patients: A Call to Action

There is now compelling evidence that respiratory muscle weakness is a highly likely consequence of prolonged mechanical ventilation. Diaphragm proteolysis is detectable within 18–69 h of controlled mechanical ventilation [6], and rapid atrophy affects respiratory muscles more frequently than limb muscles. Following at least 24 h of mechanical ventilation, respiratory muscle weakness is almost twice as prevalent as limb muscle weakness (63% vs. 34%) [2]. Even patients ventilated primarily with pressure support modes are not immune to these atrophic changes, but they are likely to have respiratory muscle weakness at the point of weaning from mechanical ventilation (e.g., 38% of predicted maximal inspiratory pressure) [7]. While “under-assistance” has been identified as a potential contributor to myotrauma [8], respiratory muscle weakness could be potentially attributable to “over-assistance” from the ventilator in pressure support mode: a recent prospective study of 231 patients in Australian ICUs identified excessive support provided in 41% of patients in pressure support mode [9]. Therefore, inspiratory muscle weakness appears to be a likely consequence of mechanical ventilation, regardless of the mode of ventilation provided.

Far from being merely an inconvenient side effect of mechanical ventilation, respiratory muscle weakness can directly affect a patient’s ventilation and ICU outcomes. Recent ultrasound studies of diaphragm thickness (a surrogate measure of inspiratory muscle strength) revealed that by day 4 of mechanical ventilation, reduced diaphragm thickness could be detected in 41% of patients [4, 10]. Reduced diaphragm thickness is associated with reduced likelihood of weaning from mechanical ventilation, higher likelihood of complications, and prolonged ICU admission [4]. Furthermore, low inspiratory muscle strength (maximal inspiratory pressure <30 cmH_2_O) at the point of extubation is associated with extubation failure, and is independently associated with 1-year mortality (hazard ratio 4.41, 95% CI 1.5–12.9) [3]. Inspiratory muscle weakness is also associated with higher ICU and hospital mortality [2]. Thus, from an ICU clinician’s perspective, inspiratory muscle weakness must be considered as a potentially treatable and reversible component in the matter of life and death.

From a patient perspective, respiratory muscle weakness typically renders patients breathless at rest, let alone during exercise [7]. Yet while ICU physiotherapists now invest considerable energy in providing early mobilization therapy to offset the impact of ICU-acquired weakness [11, 12], the respiratory muscles are frequently neglected in the rehabilitation approach [13]. Clearly respiratory muscle atrophy is an important aspect of ICU-acquired weakness, and we can no longer afford to ignore respiratory muscle rehabilitation as part of holistic recovery for ICU survivors. Identification of respiratory muscle weakness, and early commencement of targeted training, requires effective collaboration of the whole ICU multidisciplinary team, but in particular a cohesive approach between medical, nursing, and physiotherapy staff [14].

### Identifying Respiratory Muscle Weakness in ICU Patients

While researchers have used sophisticated and sometimes invasive methods to study respiratory muscle weakness in ICU patients (e.g., muscle biopsies and nerve stimulation), simple bedside measures of respiratory muscle strength do not have to be complex or invasive. For ventilator-dependent patients, features within the ventilator software can be used to obtain an approximation of maximal inspiratory pressure (e.g., “negative inspiratory force”). In this procedure, the ICU clinician coaches the patient to inhale forcefully against a “closed gate” within the system, with the resultant pressure an indication of inspiratory muscle strength. In our experience it is essential that the patient is warned that they will experience no flow of air during the attempt. While this is not a true measure of maximal inspiratory pressure, as it is not performed from residual volume (due to the presence of positive end-expiratory pressure [PEEP]), a low “negative inspiratory force” score can flag a patient for whom inspiratory muscle weakness should be suspected. Based on both our clinical experience and the data available [3], scores <30 cmH_2_O should be cause for concern.

An alternative method of inspiratory muscle strength assessment in ICU patients is the Marini method [15] where the patient exhales for 25 s through a one-way valve to reach true residual volume before maximal inhalation. This approach has been described as a strategy to obtain maximal inspiratory pressure values in ICU patients who are not responsive or cooperative [16]. However, this method has questionable inter-rater reliability in ICU patients [17], and in our clinical practice this procedure can be prohibitively stressful for patients who are conscious.

**Fig. 1:**
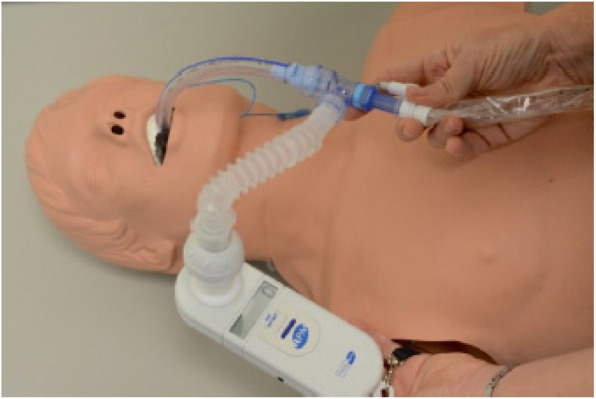
Handheld respiratory pressure manometer connected to endotracheal tube for measurement of maximal inspiratory pressure (Reproduced from [14] with permission)

Instead, we use either the method described above (i.e., ventilator-based assessment) or a handheld manometer (Fig. 1). In this latter approach, the patient is briefly disconnected from the ventilator, instructed to “empty their lungs”, and the manometer is attached to the endotracheal or tracheostomy tube via a connector. The patient then inhales maximally and the best of three attempts are recorded [18]. While maximal inspiratory pressure scores have not been found to reliably predict weaning failure [18], our experience has been that scores <30 cmH_2_O may indicate a degree of inspiratory muscle weakness which could impact on weaning and recovery. To obtain an estimate of the patient’s inspiratory muscle strength as a percentage of predicted values (that accommodate variance due to age and sex), we recommend the normalization equations provided by Evans et al. [19] (Table 1).

**Table 1:** Calculating Normal Values of Respiratory Muscle Strength [19]

Male MIP = 120 − (0.41 × age), and male MIP LLN = 62 − (0.15 × age)	
Male MEP = 174 − (0.83 × age), male MEP LLN = 117 − (0.83 × age)	
Female MIP = 108 − (0.61 × age), and female MIP LLN = 62 − (0.50 × age)	
Female MEP = 131 − (0.86 × age), and female MEP LLN = 95 − (0.57 × age)	

MIP = maximal inspiratory pressure; MEP = maximal expiratory pressure; age in years; LLN = lower limit of normal

For cooperative patients recently weaned from mechanical ventilation, measurement of maximal inspiratory pressure can be feasibly done through either the mouth or a tracheostomy using a handheld manometer [14]. However, even if ICU clinicians do not have access to this device, lack of measurement does not preclude appropriate therapy. Any patient who has recently weaned from invasive mechanical ventilation of more than 7 days duration should be regarded as at high-risk of inspiratory muscle weakness [7], and proactive targeted therapy should commence as soon as possible. This therapy is specific respiratory muscle training.

### Specific Respiratory Muscle Training: Can It Make a Difference to ICU Patients?

The availability and quality of evidence regarding inspiratory and expiratory muscle training in ICU patients differs, and we will address each in turn.

There is now convincing evidence that specific inspiratory muscle training can increase inspiratory muscle strength in ventilator-dependent ICU patients, measured as changes in maximal inspiratory pressure. Three systematic reviews and meta-analyses [20–22] have revealed that inspiratory muscle training results in higher maximal inspiratory pressure scores compared to usual care (e.g., 15 studies; pooled mean difference, 6 cmH_2_O; 95% CI, 5–8 cm [22]). A study of inspiratory muscle training in ICU patients recently weaned from mechanical ventilation [23] similarly showed significant improvements in maximal inspiratory pressure in the training group compared to the control (mean difference 11% of predicted values). Clearly, we can strengthen inspiratory muscles in ICU patients at various points in their recovery journey.

However, improvements in strength measures alone are unlikely to drive practice change. Far more relevant are the changes in patient-centered outcomes that have accompanied strength improvements in numerous studies. These include reduced duration of weaning from ventilation (five trials; pooled mean difference, 3.2 days; 95% CI 0.6–5.8 days [22]); increased likelihood of liberation from the ventilator within 28 days (71% vs. 47%) [24]; and improved quality of life [23]. While most studies have not been powered for these important outcomes, or have not measured patient-centered outcomes such as quality of life or dyspnea, these promising results indicate that the benefits of inspiratory muscle training extend beyond strength alone.

The evidence regarding the impact of expiratory muscle training in ICU patients is currently more limited. Indeed, the expiratory muscles have been described as the “neglected component” of the respiratory system in a recent comprehensive review that describes expiratory muscle physiology in ICU patients [25]. In the most recent systematic review of respiratory muscle training in ICU patients [22], four studies of expiratory muscle training (comprising 153 participants) were meta-analyzed, revealing a mean difference of 9 cmH_2_O (95% CI 5–14) in favor of the training group relative to control. However, the effect of expiratory muscle training on patient outcomes requires further exploration in an ICU context. In this light, the remainder of this chapter will focus on the implementation of inspiratory muscle training in ICU patients.

### Current Practice: Inspiratory Muscle Training in the ICU—Not All Approaches Are Equal

While we have been using inspiratory muscle training in our ICU for the past 15 years [14], we are aware that such training is a relatively new approach for many ICU clinicians. Moreover, where inspiratory muscle training is being used around the world, a wide variety of approaches is being employed, and not all of these are evidence-based. For example, a survey of French physiotherapists revealed that 83% considered controlled diaphragmatic breathing (without resistance) to be a form of inspiratory muscle training, and only 16% measured inspiratory muscle strength [26]. More recently, a meta-analysis of 28 studies of inspiratory muscle training in ICU patients described a broad range of training strategies, including strength-based and endurance-based loading (through a removable threshold device or through manipulating ventilator settings), and also more general strategies such as upper limb exercises, mobility training, and biofeedback [22]. However, to understand the strengths and limitations of the different approaches, it is essential to appreciate the importance of titratable loading with regard to muscle training.

In respiratory muscle training, “resistive loading” usually refers to patients breathing through a small aperture connector to provide a training load. A limitation of this resistive loading is that the amount of resistance (and therefore load) depends on the flow rate generated by the patient. If the patient breathes slowly enough, the load can be very low or negligible. In contrast, “threshold loading,” typically using a removable device, requires patients to generate a specific resistance as they initiate a breath to open the valve and generate flow. An important advantage of this threshold loading is that a specific, reliable, and reproducible load can be titrated and applied to the respiratory muscles [27], which can be increased over time to generate a training effect. As with all strength training, gradual increases over time are the key to muscle fiber proliferation and hypertrophy. If the load is unreliable or variable, then our ability to deliver an efficient and effective training regime is hampered, and our patients may waste valuable effort with suboptimal training. Thus, inspiratory muscle training strategies that use a titratable load are more likely to result in effective training.

Threshold loading in ICU patients is usually achieved in one of two ways: manipulating the ventilator settings (i.e., reducing the pressure trigger sensitivity, such that the patient has to increase their effort to trigger an augmented breath); or through a removable threshold device which is intermittently applied to the endotracheal tube or tracheostomy. While theoretically both approaches should result in reliable training, the outcomes are contrasting. Studies of ventilator manipulations have failed to show significant benefits in terms of either breathing muscle strength or weaning duration, despite applying training loads for up to 30 min at loads up to 40% of maximal inspiratory pressure [28]. In contrast, as outlined earlier, several studies of threshold loading (using removable devices) have demonstrated significant gains in inspiratory muscle strength and ventilator-weaning success rates [24, 29–31]. While these contrasting results may be challenging, there is a key feature differentiating the approaches: in the training with removable devices, patients must actively participate in their therapy and, if only for a short time, consciously tolerate breathing without the ventilator support. In addition to physiological adaptations, there may be a psychological dimension to this training (i.e., development of tolerance to the sensations of unsupported breathing) that would be absent in the ventilator-based approach. Future studies are needed to better elucidate the potential psychological dimensions of respiratory muscle training, but psychological factors may be key to the success of the therapy.

There is no evidence that coached deep breathing exercises (without resistance) make any difference to respiratory muscle strength or weaning outcomes in ICU patients. In fact, there is scarce evidence that deep breathing exercises (without resistance) confer any benefit in acutely unwell patients, for example, in the postoperative phase [32, 33]. Upper limb exercises and mobilization are important aspects of whole-body strengthening and rehabilitation and will also induce, via increased ventilation, an endurance type of training to the respiratory muscles. However, it is our view that ICU clinicians should be wary of classifying these as respiratory muscle strength training *per se*. Instead, focused and targeted respiratory muscle strengthening should be achieved using titratable resistance, individualized to the patient’s current level of weakness, and followed by sufficient rest periods to allow recovery as in athletic training [34]. Based on the available evidence, an international shift toward titratable loading for respiratory muscle training in ICU patients is now overdue.

### Practicalities of Inspiratory Muscle Training in ICU Patients

The most common device used to apply intermittent threshold loading in ICU patients is a simple mechanical spring-loaded one-way valve [22], where resistance can be titrated (e.g., between 9 and 41 cmH_2_O). In this approach, the patient is briefly removed from the ventilator, and the inspiratory muscle trainer is connected to the endotracheal tube or tracheostomy for training (i.e., breathing through the valve). The intensity is increased over time simply by winding the spring more tightly [14]. This device has been shown to be safe for inspiratory muscle training in selected ventilator-dependent ICU patients, with a negligible rate of adverse events [35].

Typically, this training is prescribed and supervised by the ICU physiotherapist or respiratory therapist. Whether the therapist should use a “strength” (high intensity, low repetition) or “endurance” (low intensity, longer duration) approach to prescribing training parameters is still somewhat open to debate. In the recent systematic review of inspiratory muscle training in ICU patients [22], where “strength” and “endurance” regimes were analyzed separately, both favored inspiratory muscle training relative to control groups. It could be argued that as the inspiratory muscles are primarily muscles of endurance, an endurance-based approach would be sensible [36]. However, in our experience, the highly limited window of patient effort (frequently compromised by fatigue, inattention, or delirium), coupled with the relative disadvantage of potential lung decruitment during sustained training (e.g., secondary to prolonged loss of PEEP), makes strength training a more realistic option for the ICU patient. A patient may be willing to attempt six high-resistance breaths, whereas the prospect of breathing against a low resistance for several minutes can appear prohibitively daunting. From this perspective, the best respiratory muscle training approach may be the one that the ventilator-dependent patient can successfully achieve.

Indeed, there may be psychological benefits to successfully completing short bursts of achievable work. Anecdotally, our patients often report pride and excitement when they observe, for example, that last week they could only train at 17 cmH_2_O, but this week they can train at 29 cmH_2_O. At a time in recovery when progress of any kind can feel extraordinarily slow, recovery of inspiratory muscle strength may be tangible and therapeutic at many levels. Again, this deserves more in-depth exploration from a psychological perspective.

If we are to use a strength-focused approach to training ICU patients, we should draw on the wealth of research in inspiratory muscle training in other populations (e.g., chronic obstructive pulmonary disease [COPD] [37, 38], heart failure [39], athletes [40–43]) where intensity is crucial. Early studies in COPD patients often failed to detect benefits of inspiratory muscle training where intensity was less than 30% of maximal inspiratory pressure [44, 45]. In contrast, later systematic reviews and meta-analyses which included studies with intensities great then 30% of maximal inspiratory pressure were more favorable not just for inspiratory muscle strength but also for exercise tolerance and quality of life [37, 46]. In athletes, inspiratory muscle training intensity is typically prescribed between 50% and 80% of maximal inspiratory pressure, across endurance sports such as swimming [42], cycling [41], rowing [40], and running [43]. With high-intensity training, athletes improve not just inspiratory muscle strength but frequently exercise performance as well (e.g., 2.6% increase in 25 km cycling time trial performance [41]; 3.5% increase in 6-min rowing time trial performance [40]). In studies of inspiratory muscle training in ICU patients, high-intensity training (6 repetitions per set, >50% of maximum, 30 breaths per day) has resulted in improvements in inspiratory muscle strength [24, 30], and in the postweaning period has improved quality of life [23]. Therefore, wherever possible, a high-intensity approach to strength training should be used to optimize inspiratory muscle training in ICU patients.

Acknowledging the relative advantages of the simple mechanical threshold device (including its low cost and accessibility), we have experienced some limitations of this device in an ICU context. First, the floor effect of this spring-loaded device can be problematic for a patient who is profoundly weak (e.g., maximal inspiratory pressure <18 cmH_2_O). If the lowest setting of the device is only 9 cmH_2_O, patients may struggle to open the valve at its lowest setting. Second, we have also noted a ceiling effect. Toward the end of training, several of our patients have comfortably exceeded the 41 cmH_2_O upper limit of the device and would be capable of training at much higher intensities. While for most patients this might not be necessary, for those returning to a more active lifestyle, continuing to improve their inspiratory muscle strength may be a vehicle to better tolerance of endurance exercise. To better suit the needs of our ICU patients, we require devices with a broader training spectrum.

### Emerging Strategies for Inspiratory Muscle Training in ICU Patients

**Fig. 2:**
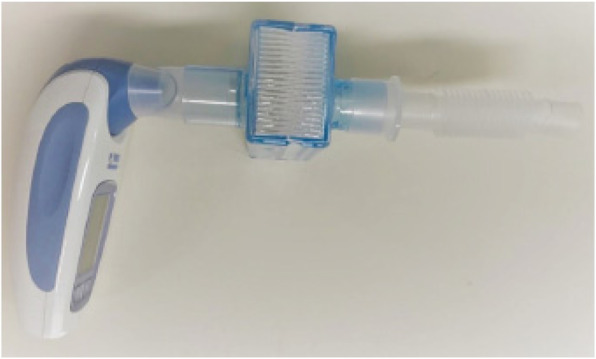
Attachment of electronic inspiratory muscle training device to filter and connector

In the past few years there have been crucial developments in the sophistication of inspiratory muscle training devices. Electronic devices provide a much wider training spectrum (e.g., from 1 to 200 cmH_2_O), and although they are more expensive than the disposable spring-loaded device, they have other advantages, including the capacity to measure performance within the device (e.g., maximal inspiratory pressure, tidal volume, work and energy of breathing during the training session). As a handheld, chargeable device, they are ideally suited for bedside treatment in the ICU and can be adapted to interface with either a tracheostomy or an endotracheal tube via a simple connector (Fig. 2).

**Fig. 3:**
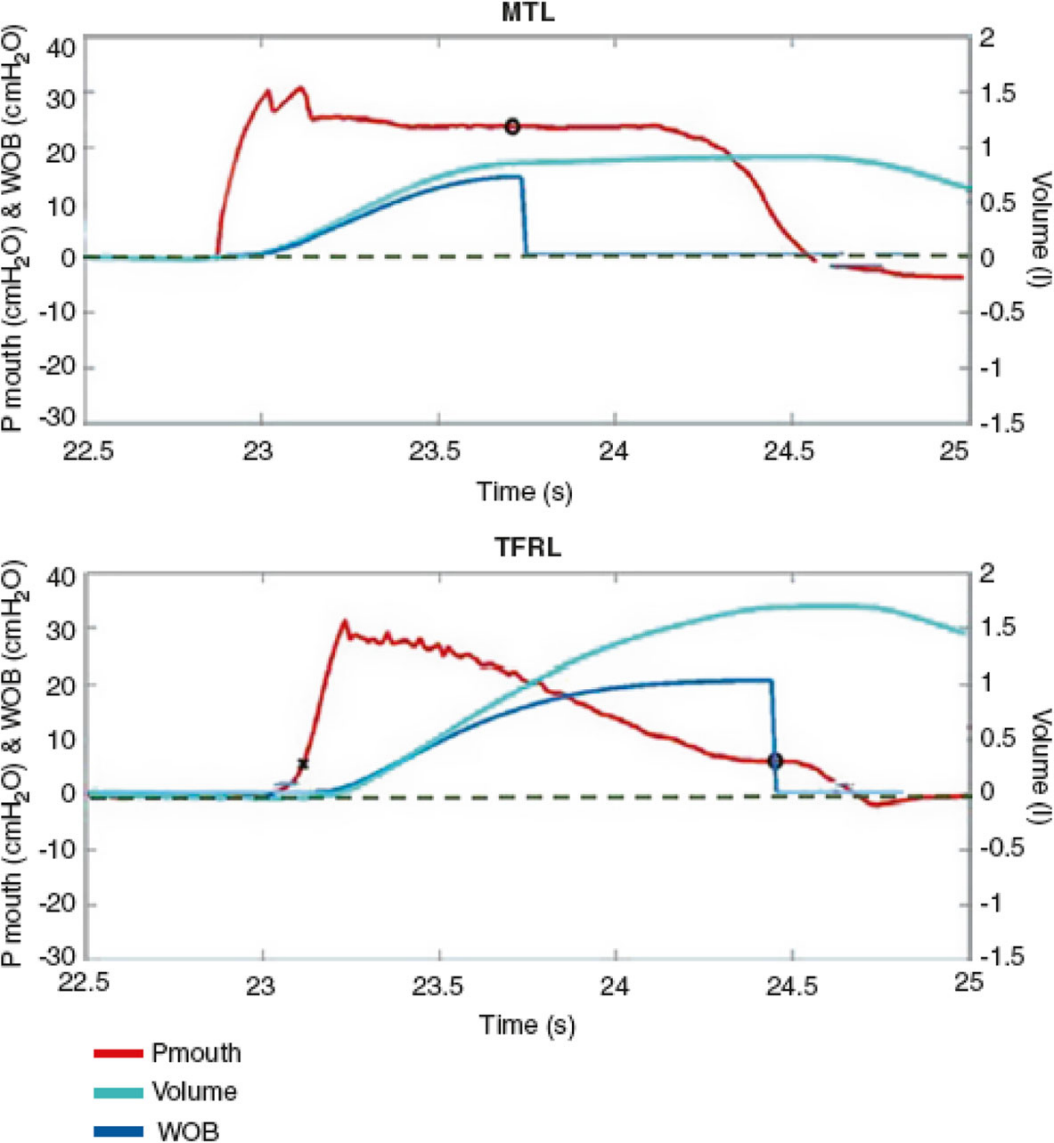
A single respiratory cycle, comparing mechanical threshold loading (MTL) with tapered flow resistive loading (TFRL) in a ventilator-dependent patient. *Pmouth* pressure at the mouth, *WOB* work of breathing

The most important difference in the design of these devices is the incorporation of a tapered flow resistance load. The advantages of a tapered flow resistance load have been well-described in patients with COPD [47] but may also be advantageous for ICU patients. Briefly, whereas a traditional threshold load requires the patient to generate a preset pressure, beyond which they can “coast” through the rest of the breath, the tapered flow resistance load provides a tapered load beyond the threshold point, meaning that patients continue to work throughout the duration of the breath, rather than “coasting.” The result is that for each breath at the specified intensity, the patient generates more work (at a guaranteed achievable resistance). Thus, the total workload (and therefore potential training effect achievable) is considerably higher with tapered flow resistance load compared to traditional threshold loading. The following graph from a study of tapered flow resistance load in a ventilator-dependent ICU patient captures this difference (Fig. 3).

**Fig. 4:**
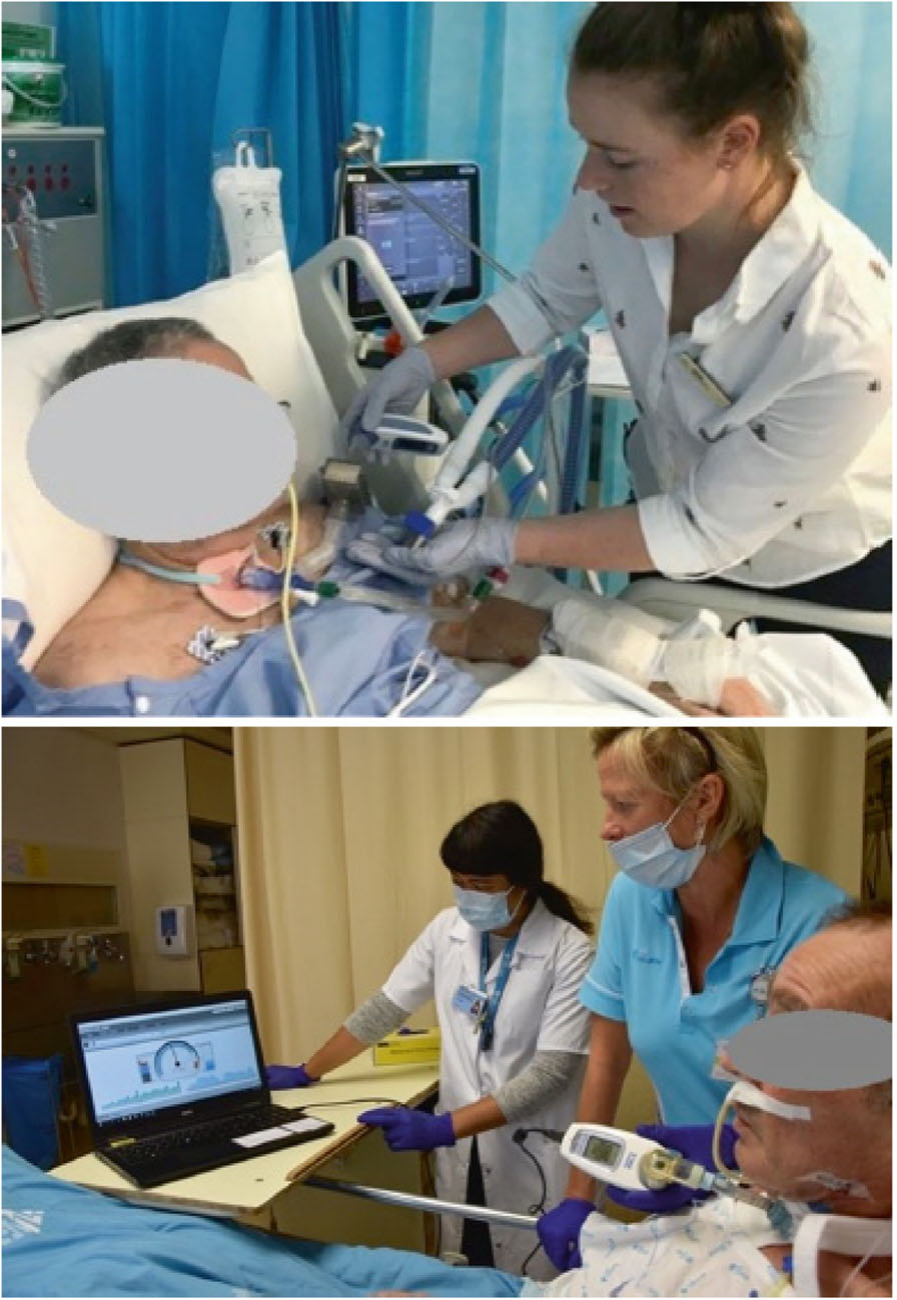
Tapered flow resistive loading inspiratory muscle training in a ventilator-dependent patient (upper panel). Visual feedback provided during tapered flow resistive loading in a ventilator-dependent patient (lower panel)

So far, there has been one randomized trial of tapered flow resistance load inspiratory muscle training in ICU patients, where it was compared with a sham treatment of intermittent nebulization [29]. Although this was a small study, capturing 21 patients, the results were encouraging: while the sham intervention group had a mean ventilatory weaning time of 9.4 days, the tapered flow resistance load training group’s mean weaning time was 3.5 days, and this difference was statistically significant (*p* = 0.0192) [29]. Clearly, we need more studies in different patient cohorts to confirm these findings and elucidate the optimal training approach. Another major randomized trial is underway, using tapered flow resistance load and comparing both strength and endurance approaches [48]. We await the results of this study with keen interest. Meanwhile our clinical experience of tapered flow resistance load training (Fig. 4) is that it is well-tolerated by ICU patients and readily captures considerably more data for analysis by the treating clinicians (including maximal inspiratory pressure, work of breathing, tidal volume).

Furthermore, this new technology can provide visual feedback of the training on a computer screen (Fig. 4 lower panel). This information allows better guidance of the training by the physiotherapist, while the visual feedback on the screen stimulates the patient to achieve large tidal volumes to ensure loading of the inspiratory muscles over full range of motion.

### Barriers to Respiratory Muscle Rehabilitation in ICU Patients

Potential contraindications to inspiratory muscle training in ICU patients have been identified and include pre-existing neuromuscular disease, hemodynamic instability (arrhythmia, decompensated heart failure, coronary insufficiency), hemoptysis, use of any type of home mechanical ventilatory support prior to hospitalization, any skeletal pathology that impairs chest wall movements such as severe kyphoscoliosis, congenital deformities or contractures, poor general prognosis, or anticipated fatal outcome [48].

One major barrier to effective inspiratory muscle training in ICU patients is that this approach requires the patient to be awake and actively participating in their training. Patients need to be capable of understanding and tolerating an increased resistance for short periods, without being overly distressed by it. If patients are too sedated, they cannot benefit. In the landscape of reducing sedation to facilitate early rehabilitation in the ICU [1], this provides yet another imperative to minimize (or eliminate) sedation as early as possible.

**Fig. 5:**
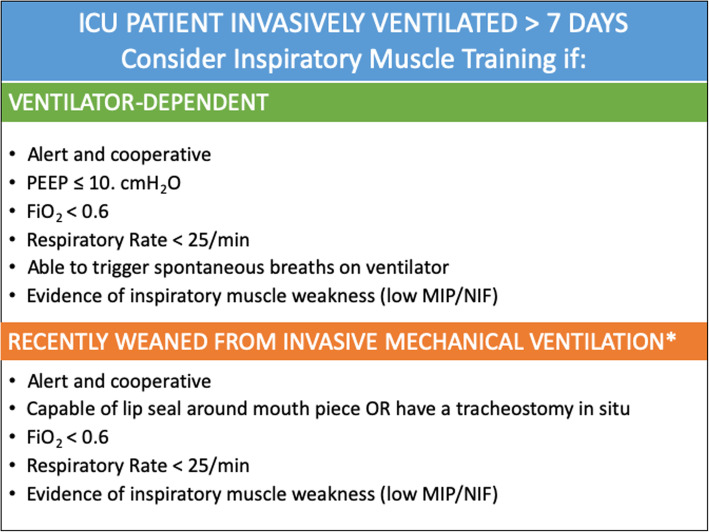
Criteria for suitability for inspiratory muscle training for ICU patients. *PEEP* positive end-expiratory pressure, *FiO*_*2*_ fraction of inspired oxygen, *RR* respiratory rate, *MIP* maximum inspiratory pressure, *NIF* negative inspiratory force (measured on the ventilator). *Recently weaned means independently breathing 24 hours per day without any invasive ventilatory support. (Reproduced from [14] with permission)

In our recent practice guideline for inspiratory muscle training in ICU patients [14], we outlined criteria for suitability (Fig. 5) and identified patients for whom inspiratory muscle training is not appropriate, including those who require high levels of PEEP (e.g., >15 cmH_2_O), those with high respiratory rates (e.g., >25 breaths per minute) or deteriorating respiratory or cardiovascular stability. From a purely practical perspective, inspiratory muscle training is also not feasible in patients experiencing extreme pain or dyspnea, and these will need to be addressed to facilitate effective treatment.

It is likely that some ICU patients will not be able to participate in inspiratory muscle training while ventilator-dependent, due to a combination of factors that may include sedation, delirium, or physiologic instability. Given the very high likelihood that these patients will have significant respiratory muscle weakness when they are eventually weaned from the ventilator, is there any advantage to commencing respiratory muscle training after liberation from the ventilator? The good news is that these patients can still benefit from training in the postweaning period. In a study of inspiratory muscle training in recently weaned ICU patients (invasively ventilated for 7 days or more), 70 patients were randomized to either usual care or additional daily threshold-based inspiratory muscle training [23]. Two weeks of daily training improved inspiratory muscle strength (maximal inspiratory pressure) as well as quality of life. Patients were most likely to benefit if they had at least moderate inspiratory muscle strength (28 cmH_2_O or more) [49]. Therefore, a targeted approach to inspiratory muscle training in recently weaned ICU patients appears to be a worthwhile investment.

### Future Directions for Respiratory Muscle Rehabilitation in ICU Patients

While inspiratory muscle training is effective in strengthening inspiratory muscles and accelerating ventilator weaning in ICU patients, we are yet to elucidate the optimal training parameters. Current and future studies will guide clinicians regarding the relative value of strength or endurance approaches to training, but in the short term it appears that strength training (high-intensity low repetition loading) is feasible and effective, both for ventilator-dependent patients and in the postweaning phase of recovery. While mechanical threshold loading can be effective in patients with moderate inspiratory muscle weakness, electronic inspiratory training may be better suited to profoundly weak ICU patients.

Although this chapter has focused on the physical and physiological aspects of respiratory muscle rehabilitation, future research needs to also consider the contribution of psychological factors to ventilatory weaning and rehabilitation. As has been described most eloquently with respect to patients with COPD, dyspnea is best understood as a complex and individual phenomenon, highly modified by emotional, cognitive, and contextual factors [50]. In an ICU environment, these factors could include fear and anxiety, as well as cognitive challenges around attention, catastrophizing, and perceived lack of control. A better understanding of the psychological dimension of dyspnea in ICU patients could further inform our approach to optimized ventilatory weaning and respiratory muscle rehabilitation. We hope that future studies of ICU patients will incorporate these patient-centered perspectives and shape our understanding of how best to facilitate holistic recovery.

## Conclusion

Early and proactive rehabilitation of the respiratory muscles is feasible and effective in ICU patients. As respiratory muscle weakness clearly affects outcomes within and beyond the ICU, the multidisciplinary team should implement targeted and individualized training of respiratory muscles to optimize patient recovery. Inspiratory muscle training can facilitate ventilator liberation, while potentially improving patients’ quality of life. Given the return on investment of this relatively low-cost therapy, respiratory muscle rehabilitation should be considered a priority in the modern approach to the management of ICU-acquired weakness.

## Data Availability

The data that support the findings of this manuscript are available from the authors upon reasonable request.
